# Effects of computer reminders on complications of peripheral venous catheters and nurses’ adherence to a guideline in paediatric care—a cluster randomised study

**DOI:** 10.1186/s13012-016-0375-9

**Published:** 2016-01-27

**Authors:** Ulrika Förberg, Maria Unbeck, Lars Wallin, Eva Johansson, Max Petzold, Britt-Marie Ygge, Anna Ehrenberg

**Affiliations:** 1 School of Education, Health and Social Studies, Dalarna University, Falun, Sweden; 2 Department of Women’s and Children’s Health, Karolinska Institutet, Karolinska University Hospital, Elevhemmet H2:00, 171 76, Stockholm, Sweden; 3 Division of Orthopaedics, Department of Clinical Sciences, Danderyd Hospital, Karolinska Institutet, Stockholm, Sweden; 4 Department of Neurobiology, Care Sciences and Society, Karolinska Institutet, Stockholm, Sweden; 5 Centre for Applied Biostatistics, Department of Medicine, Sahlgrenska Academy, University of Gothenburg, Gothenburg, Sweden; 6 Karolinska Institutet, ICHAR, Stockholm, Sweden

**Keywords:** Peripheral venous catheter, Clinical decision support system, Computer reminder, Paediatric, Clinical practice guideline, Cluster RCT, Implementation, Context

## Abstract

**Background:**

Reminder systems in electronic patient records (EPR) have proven to affect both health care professionals’ behaviour and patient outcomes. The aim of this cluster randomised trial was to investigate the effects of implementing a clinical practice guideline (CPG) for peripheral venous catheters (PVCs) in paediatric care in the format of reminders integrated in the EPRs, on PVC-related complications, and on registered nurses’ (RNs’) self-reported adherence to the guideline. An additional aim was to study the relationship between contextual factors and the outcomes of the intervention.

**Methods:**

The study involved 12 inpatient units at a paediatric university hospital. The reminders included choice of PVC, hygiene, maintenance, and daily inspection of PVC site. Primary outcome was documented signs and symptoms of PVC-related complications at removal, retrieved from the EPR. Secondary outcome was RNs’ adherence to a PVC guideline, collected through a questionnaire that also included RNs’ perceived work context, as measured by the Alberta Context Tool. Units were allocated into two strata, based on occurrence of PVCs. A blinded simple draw of lots from each stratum randomised six units to the control and intervention groups, respectively. Units were not blinded. The intervention group included 626 PVCs at baseline and 618 post-intervention and the control group 724 PVCs at baseline and 674 post-intervention. RNs included at baseline were 212 (65.4 %) and 208 (71.5 %) post-intervention.

**Results:**

No significant effect was found for the computer reminders on PVC-related complications nor on RNs’ adherence to the guideline recommendations. The complication rate at baseline and post-intervention was 40.6 % (95 % confidence interval (CI) 36.7–44.5) and 41.9 % (95 % CI 38.0–45.8), for the intervention group and 40.3 % (95 % CI 36.8–44.0) and 46.9 % (95 % CI 43.1–50.7) for the control. In general, RNs’ self-rated work context varied from moderately low to moderately high, indicating that conditions for a successful implementation to occur were less optimal.

**Conclusions:**

The reminders might have benefitted from being accompanied by a tailored intervention that targeted specific barriers, such as the low frequency of recorded reasons for removal, the low adherence to daily inspection of PVC sites, and the lack of regular feedback to the RNs.

**Trial registration:**

Current Controlled Trials ISRCTN44819426

## Background

A majority of hospitalised children require a peripheral venous catheter (PVC) for intravenous treatment, but indwelling PVCs might lead to complications such as infection, infiltration, occlusion, and/or thrombophlebitis, that can affect the child’s health and wellbeing [1]. The insertion and management of PVCs are very common procedures that registered nurses (RNs) in Sweden perform on a daily basis. Maintaining optimal function of PVCs in paediatric patients is essential, as re-insertion is often highly stressful for the patients [2]. As PVCs in paediatric care should be replaced when clinically indicated [3], the importance of RNs’ adherence to guidelines such as the recommended daily inspections of PVC sites is important.

One of the most consistent findings from clinical and health services research is the failure to implement research into practice and policy [4]. Clinical practice guidelines (CPGs) are commonly used as a strategy to overcome this gap, but the assumption that a CPG will implement itself is long gone, as there are several factors to consider for its implementation [5]. Successful implementation can, according to the Promoting Action on Research Implementation in Health Services (PARIHS) framework, be understood as a function of the relationship between evidence, context, and facilitation [6–8]. The framework suggests that successful implementation is more likely to occur when evidence and context are considered high. Context comprises three dimensions: culture, leadership, and evaluation [9]. The rationale for choosing the PARIHS framework is that it describes influential factors involved in implementing evidence-based practice in nursing care. Furthermore, the framework has been used in several studies, and the dimensions have been validated in a number of studies [10]. However, PARIHS has also been criticised, for example, it is not clear what role individuals play as part of the interaction between evidence and context [11] and that the influence of staff turnover is not considered [12].

There are several strategies used for disseminating and implementing CPGs such as distribution of educational material, audit and feedback, and tailored interventions. A review on the effectiveness and efficiency of guideline dissemination and implementation strategies for physicians published in 2004 showed that a majority of the strategies resulted in modest to moderate improvements in patient care [4] but that the most promising strategy was in the form of reminders. Computerised decision support systems (CDSSs) are electronic systems designed to aid directly in clinical decision-making by generating recommendations [13]. According to Choi and colleagues [14], CDSS can be categorised into three groups: (1) reminders, notifications, alerts, or warnings designed to regularly remind healthcare workers of, e.g. assessments, monitoring, and scheduled care; (2) point of care guidelines, which provide necessary information and guidelines for certain activities or offers automatic care plans or checklists based on individual patient data; and (3) references for information/guidelines, which enable health care professionals to search for necessary information. Computer reminders are defined as “patient or encounter specific information, provided via a computer console either visually or audibly, which is designed or intended to prompt a health care professional to recall information usually encountered through their general medical education, in the medical records or through interaction with peers, and so remind them to perform or avoid some action to aid individual patient care” [15, p. 4]. There are no systematic reviews on the effect of computer reminders on nursing performance or patient outcomes; there are however systematic reviews on CDSS which have shown that CDSS effectiveness in nursing practice is still inconsistent and that further studies are needed in order to identify in which contexts they are most effective [16, 17]. Studies in recent years of CDSS have shown improvement in nursing recording [18] and patient outcomes [19], but single studies of computer reminders have shown effect on nurses’ adherence to CPGs [20] and decreased omissions in nursing care [21].

To summarise, computer reminders have shown some effect on physicians’ behaviour and outcomes of care, but knowledge is lacking on the effect on RNs’ behaviour and patient outcomes and how contextual factors might influence the implementation. The main aim of this cluster randomised study was to investigate the effects of implementing a CPG for PVCs in paediatric care in the format of reminders, integrated in the electronic patient record (EPR), on PVC-related complications, and on RNs’ self-reported adherence to the guidelines. An additional aim was to study the relationship between contextual factors and the outcomes of the intervention. As the computer reminders could only be implemented at unit level, a cluster randomised design was chosen.

## Methods

### Setting

The study was conducted at a paediatric hospital, a division within a large urban university hospital in Sweden. The paediatric hospital is located at three sites and admits patients from 0 to 18 years of age. At the time of the study, the hospital had a capacity of 245 beds and consisted of 19 inpatient units and approximately 940 RNs were employed.

Insertion and management of PVCs was an area identified for quality improvement at the hospital, and a CPG was developed in 2010. The CPG was developed by RNs with expert knowledge of PVCs in paediatric care and was based on critical appraisal of published research as well as RNs’ professional experiences. The CPG contained recommendations on PVC management, for example, it suggested indications for insertion, sizes, and insertion sites, as well as instructions for insertion, management, and removal; additionally, it contained information concerning PVC-related complications. The CPG was introduced both by the hospital through information at the nurse managers’ monthly meeting and by publication on the intranet, which was accessible via the computers in nursing stations.

The first transition from paper-based patient records to an EPR system took place in 1997, and the current system was introduced in 2005. At the time of the study, all patient data were recorded in the EPR and there were computers at all nursing stations. A template for recording PVCs in a structured and standardised way was introduced at the hospital in 2009 as an option for documenting in the record notes [22]. The fields concerning PVC insertion in the template was mandatory, meaning that the template could not be closed until these fields were completed. Several fields contained drop-down options where only one option could be selected (Table 1). Mouse pads containing information on how to document PVCs in the template were handed out on intervention and control units at the start of the intervention.

**Table 1:** The content of the reminders integrated into the PVC template in the EPR

Structure and content of the PVC template in the EPR (all units)		Reminders based on recommendations from the CPG (intervention units)
Insertion date^a^	yyyy-mm-dd	Reminder! Disinfect your hands and forearms; use disposable gloves. Disinfect the insertion area thoroughly. Fixate the PVC well, making sure that the insertion site can be observed.
Reason for insertion^a,b,c^	Intravenous therapy/preparation for surgery or examination/risk that the patient can deteriorate/no obvious reason/other reasons (free text)	Reminder! Always use aseptic technique when managing the PVCs and the catheter system.
Insertion attempts^a,b,c^	1/2/3/4/5/other numbers (free text)	
Side^a,b^	Right/left	
Size^a,b^	26G (purple)/24G (yellow)/22G (blue)/20G (pink)/18G (green)/17G (white)/16G (grey)	Reminder! Choose a short and thin PVC as possible.
Site^a,b^	Hand/wrist/forearm/bend of arm/groin/foot/ankle/scalp/lower part of the leg/other insertion sites (free text)	
Removal date	yyyy-mm-dd	Reminder! Document the reason for removal.
Removal cause^b^	Completed treatment/occlusion/pain/suspicion of infection/thrombophlebitis/thrombosis/infiltration/other reasons for removal (free text)	
Inspection day, 1–10^b,c^	No signs or symptoms of complications/erythema/swelling/heat/pain/pain at palpation/pus or liquid/other signs/symptoms (free text)	Reminder! Remove the outer dressing, inspect the insertion site, and flush the PVC. Ask the patient for PVC-related pain and pain at palpation. Assess whether the PVC should remain in situ.

^a^Mandatory fields for recording

^b^Drop-down options, with a free text option

^c^These options were introduced into the EPR system during the second data collection

### Intervention

The reminders were integrated in the PVC template at the intervention units over 3 months. Thus, RNs at the intervention units could not be blinded. The reminders were developed in collaboration between the researchers and RNs who were experts in EPRs. They were designed as speech bubbles that appeared on the screen for 20 s when the cursor was moved over the template. The reminders had a font and a background colour that deviated from the rest of the template. They consisted of five recommendations originating from the CPG for PVC management: disinfection of hands, use of disposable gloves, fixation of and choice of PVC size, inspection of insertion site, and documentation of removal cause (Table 1). These recommendations were selected, as adherence to them was presumed to have the greatest impact on patients with PVCs [3].

### Outcomes

The primary outcome was recorded occurrence of PVC-related complications at removal assessed by the bedside RN. Data was collected from the PVC template at baseline and post-intervention. The secondary outcome was RNs’ self-reported adherence to the CPG recommendations: disinfection of hands, usage of disposable gloves, and daily inspection of PVC site measured through a questionnaire, handed out at baseline and post-intervention.

### Sample size calculation and randomisation

In a record review performed at 14 inpatient units at the paediatric hospital in 2009, we found 87 PVCs in 147 patients, which yielded an expected number of six PVCs on each unit over 5 days. The proportion of recorded PVC-related complications in the EPR discovered during the record review was 34 %. The record review indicated that it was appropriate to split participating units into two strata based on the expected prevalence of PVCs. Eight units (surgery, cardiology, orthopaedic, neurology, two infection units, advanced homecare, and neonatal intensive care unit) were in the strata of high occurrence of PVCs, while four units (oncology, haematology, and two neonatal units) were in the strata of lower occurrence. An expected decrease of complications for the intervention units was set at 6–10 % based on findings from a systematic review [23].

A sample size calculation was performed to estimate the number of recorded PVCs required for this study [24]. The sample size of 1213 PVCs, about 100 PVCs per unit, was calculated to be necessary to have 80 % power to detect a difference in complications of odds ratio (OR) = 1.5 between control and intervention groups at a significance level of 5 % and an intra-cluster coefficient of 0.001 on the unit level. An earlier study [22] on the accuracy and completeness of PVC recordings showed that a majority of observed complications were not recorded in the EPR. On this basis, a total of 1213 PVCs from the EPR were included in each group at both baseline and post-intervention in order to secure power. The randomisation was carried out by a third person through a simple draw of lots from each of the strata, allocating six units to the control and intervention groups, respectively.

### Sample

#### Units, PVCs, and patients

**Fig. 1:**
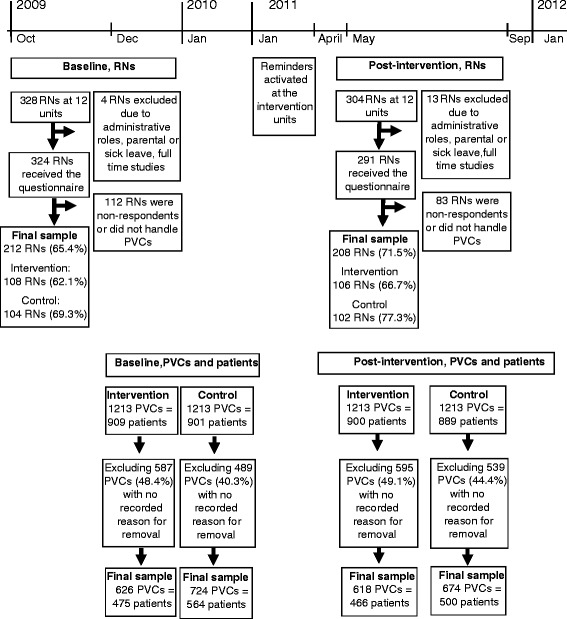
Flow chart for inclusion RNs, PVCs, and patients at baseline and post-intervention

A requirement for inclusion of inpatient units was that they had access to the PVC template in the EPR system to document PVCs during the study period, which excluded seven of the hospital’s 19 inpatient units. One unit in the intervention group changed to another EPR system 60 days after the introduction of the reminder. This unit was therefore only included for 60 days post-intervention and, consequently, also 60 days at baseline. The PVCs should have been inserted and documented in the PVC template at any of the 12 units at baseline (before 11 January 2011, the introduction date of the reminders) and post-intervention (after 1 April 2011). Each PVC was counted as one case and data were retrieved from the EPR system retrospectively at baseline and prospectively post-intervention. PVCs with no complete recording of reason for removal were excluded. Figure 1 describes the inclusion and exclusion of PVCs and patients. The final sample for PVCs in the intervention group was 626 at baseline and 618 post-intervention; in the control group, 724 PVCs were included at baseline and 674 post-intervention. As some patients had more than one PVCs, the final sample of patients at baseline was 475 in the intervention group and 466 in the control group; at post-intervention, there were 564 in the intervention group and 500 in the control group (Fig. 1).

#### Registered nurses

Inclusion criteria for RNs were that they worked at one of the 12 inpatient units at the time for data collection. The data collection periods at baseline stretched from October 2009 to January 2010 and post-intervention from May to September 2011. Exclusion criteria were if RNs had only administrative duties or were on parental leave, sick leave, or doing full-time studies during the data collection periods. Figure 1 describes the inclusion and exclusion of RNs. The final sample of RNs at baseline in the intervention group was 108 (62 % response rate) and 104 (69 % response rate) for the control group, while the final sample post-intervention was 106 RNs (67 % response rate) for the intervention group and 102 (77 % response rate) for the control group, as seen in Fig. 1.

### Data collection

#### PVC-related complications, PVCs, and patients

Data concerning PVCs and patient demographics were collected from the EPR system with a support from a blinded data collector at the County Council’s IT and EPR administration. The PVC template included eight different drop-down options for removal of PVCs, as seen in Table 1. When the removal cause “other reasons for removal” was used but an explanatory text was missing or when there were incomplete demographics, data records were manually reviewed for a more detailed information.

#### Registered nurses adherence, demographics, and context

Data concerning RNs’ adherence, demographics, and their perceptions of the unit context were collected through a questionnaire. Adherence to the CPG recommendations was measured through nine items regarding RNs’ management of PVCs. These items were based on the hospital’s CPG for PVCs; three of the items were selected for the analyses as adherence to them was presumed to have the greatest impact on patients with PVCs [3]: How often do you disinfect your hands with alcohol-based products before managing PVCs? How often do you use disposable gloves when managing PVCs? How often do you perform daily inspection of the PVCs’ insertion sites? The items had a five-point Likert response scale: never, rarely, occasionally, frequently, and always. One item addressed whether the RNs knew about the hospital’s CPGs for venous catheters, with the response alternatives yes or no. The demographic data for RNs can be seen in Table 2. RNs work context was measured using the Alberta Context Tool (ACT), conceptually framed by the PARIHS framework and designed to assess context within complex healthcare settings with the assumption that context has a central influence on healthcare professionals’ use of knowledge [25]. The ACT adds five additional dimensions to the PARIHS elements of context (leadership, culture, and evaluation), namely, information-sharing interactions, information-sharing activities, information-sharing social processes, structural and electronic resources, and organisational slack (representing human resources, space, and time). In the present study, we used the data that referred to the three dimensions of context based on the PARIHS framework: *Leadership* reflecting emotionally intelligent leadership, *Culture* describing a supportive work culture, and *Evaluation* describing the use of data to provide feedback on the unit’s performance (e.g. infection rates). Each dimension included six items and was answered on a five-point Likert scale: strongly disagree, disagree, neither agree nor disagree, agree, and strongly agree. The ACT version used in this study is modified specifically for paediatric acute care settings [26] and has previously been used to investigate the influence of organisational context on RNs use of research in Canadian paediatric hospitals [27–30]. Permission to use the translated Swedish ACT version [31] was granted by the developers. The ACT has been tested for validity and reliability in several studies [32] with a Cronbach’s α for leadership, culture, and evaluation ranging between 0.72–0.91 [25] and 0.74 and 0.90 in a Swedish paediatric setting [33].

**Table 2:** Background characteristics for PVCs, patients and RNs

	Intervention		Control	
PVCs	Baseline (*n* = 626)	Post-intervention (*n* = 618)	Baseline (*n* = 724)	Post-intervention (*n* = 674)
Median for PVC days (range)	2 (1–14)	2 (1–12)	2 (1–12)	3 (1–14)
Mean, PVC days (standard error)*	2.6 (0.1)	2.7 (0.1)	2.8 (0.1)	2.9 (0.1)
Size, *n* (%)				
26G	142 (22.8)	121 (19.6)	234 (32.4)	240 (35.6)
24G	307 (49.3)	338 (54.7)	365 (50.5)	296 (43.9)
22G	154 (24.7)	151 (24.4)	109 (15.1)	115 (17.1)
20G	20(3.2)	6 (1.0)	13 (1.8)	19 (2.8)
18G	–	2 (0.3)	1 (0.1)	4 (0.6)
17G	–	–	1 (0.1)	–
Missing	3 (0.5)	–	1 (0.2)	–
Site, *n* (%)				
Hand	229 (36.8)	228 (37.0)	269 (37.2)	254 (37.7)
Wrist	21 (3.4)	23 (3.7)	24 (3.3)	18 (2.7)
Forearm	17 (2.7)	22 (3.6)	16 (2.2)	19 (2.8)
Bend of the arm	208 (33.4)	222 (36.0)	217 (30.0)	187 (27.7)
Upper part of the arm	–	1 (0.2)	–	–
Toe	–	–	–	1 (0.1)
Foot	92 (14.8)	63 (10.2)	121 (16.7)	110 (16.3)
Ankle	20 (3.2)	23 (3.7)	20 (2.8)	10 (1.5)
Lower part of the leg	3 (0.5)	4 (0.6)	–	–
Groin	–	–	2 (0.3)	4 (0.6)
Neck	1 (0.2)	–	–	–
Head	32 (5.1)	31 (5.0)	55 (7.6)	71 (10.5)
Missing	3 (0.5)	1 (0.2)	–	–
Patients/unique admissions	Baseline (*n* = 475)	Post-intervention (*n* = 466)	Baseline (*n* = 564)	Post-intervention (*n* = 500)
Median age, years (range)	3.0 (0–19)	3.5 (0–25)	2.0 (0–19)	1.5 (0–19)
Female, *n* (%)	226 (47.6)	201 (43.1)	253 (44.9)	209 (41.8)
Male, *n* (%)	249 (52.4)	265 (56.9)	311 (55.1)	291 (58.2)
Acute admission, *n* (%)	340 (71.6)	331 (71.0)	394 (69.9)	331 (66.2)
Median length of stay, days (range)	5 (1–100)	5 (1–100)	4 (1–95)	4 (1–255)
RNs	Baseline (*n* = 108)	Post-intervention (*n* = 106)	Baseline (*n* = 104)	Post-intervention (*n* = 102)
Median age, years (range)	36 (23–59)	36 (22–61)	35 (24–62)	35 (24–62)
Female, *n* (%)	102 (94.4)	100 (97.1 )	102 (99.0)	100 (98.0)
Median years since nursing certificate (range)	9.0 (1–40)	7.0 (0–40)	7.0 (1–42)	6.0 (0–39)
Median years at current unit (range)	3.1 (0–32)	2.7 (0–15)	3.7 (0–40)	2.0 (0–25)
Employment				
Full-time, *n* (%)	64 (60.9)	72 (70.6)	58 (55.8)	48 (48.0)
Part-time, *n* (%)	41 (39.0)	30 (29.4)	46 (44.2)	52 (51.0)
Educational level				
Basic, *n* (%)	53 (52.0)	56 (54.0)	62 (59.6)	67 (66.0)
Advanced, *n* (%)	49 (48.0)	47 (46.0)	42 (40.4)	34 (34.0)
Awareness of the CPGs, *n* (%)	74 (68.5)	72 (67.9)	61 (58.7)	79 (77.5)

*Test of difference in the mean PVC days from baseline to post-intervention between the groups, *p* = 0.88

The questionnaire was placed in the RNs’ mailboxes throughout the hospital. A cover letter providing information about the study and a prepaid envelope addressed to a registration bureau accompanied the questionnaire. In addition, all RNs received an e-mail informing them about the study. Three rounds of reminders were distributed via e-mail to non-responders.

### Ethical approval

Ethical approval was granted by the Regional Ethical Review Board in Uppsala (no. 2008/360). Permission to collect data through the electronic patient record system was granted by the director of the paediatric hospital and by each department’s chairman.

The RNs were informed about the voluntary nature of participation and the confidential handling of research data. Informed consent was assumed if the questionnaire was returned.

### Data analysis

#### PVC-related complications

PVCs can be removed due to complications or for elective reasons. Complications comprised infiltration including extravasation, occlusion, signs and symptoms of thrombophlebitis, PVCs accidentally removed, suspicion of infection, and wounds. Elective reasons included completed treatment, the patient being bothered by the PVC, PVCs re-sited in connection with blood sampling or change to central venous access device, and other reasons (e.g. location dependent, been inserted too long, blood at insertion site). Exact 95 % confidence interval for proportions based on the binomial distribution is provided. Differences between intervention and control groups from baseline to post-intervention in reasons for removals of PVCs were analysed using logistic regression analysis. An interaction effect of being in the intervention group post-intervention was used for assessing the intervention effect. A result of an increased adherence to the CPG could have been an increased number of PVC days; thus, the difference of PVC mean days from baseline to post-intervention was analysed using linear regression.

#### Registered nurses’ adherence and demographics

The scorings of RNs’ adherence to the CPG were dichotomized into always versus not always (representing never, rarely, occasionally, and frequently), as the CPG prescribed that the recommendations should always be performed to ensure patient safety. RNs’ educational background and employment were divided into basic education (certificate or bachelor) versus advanced (master or specialist post-graduate qualification). Categorical variables are presented as frequency counts and percentage. Symmetrically distributed data are presented as means, and standard deviation and asymmetrically distributed data are presented as median and range. Statistical analysis of the effect of the intervention on RNs’ adherence was performed in the same way as for PVC-related complications.

#### Context

RNs’ individual mean scores for the ACT dimensions leadership, culture, and evaluation were calculated. The mean scores of each dimension for the intervention and control group were calculated by adding RNs’ individual mean scores per dimension divided by the number of RNs per group. RNs’ mean scores for each context dimension were used to categorise their scores into high and low, with a cut-off set at >3.5 (high) and ≤3.5 (low) [27]. RNs’ scorings were then allocated into four context groups: high context, requiring high on all three context dimensions; moderately high context, high on two context dimensions and low on one; moderately low context, high on one and low on the two remaining; and low context, low on all three context dimensions [34, 35]. Fisher’s exact test was applied between the four context groups for the intervention and control group, respectively, with statistical significance set at *p* < 0.05.

## Results

The most common PVC sizes in both groups were 24 gauge, followed by a larger size in the intervention group and a smaller size in the control group. The most frequently used PVC site in both groups was the hand followed by the bend of the arm, foot, and head. There was no significant difference in mean PVC days from baseline to post-intervention between the groups (Table 2). A majority of the admissions in both groups were acute and male patients. The median length of stay was 5 days in the intervention group and 4 days in the control group. The median age for patients in the intervention group was 3.0 years at baseline and 3.5 years post-intervention, and in the control group, 2.0 years at baseline and 1.5 years post-intervention.

The mean number of RN respondents per unit was 17 in both groups, and the percentage ranged from 55.2 to 72.0  % at the intervention units and 52.6 to 88.9 % at the control units. The median age for RNs in the intervention group was 36 years at baseline and 35 years in the control group. The percentage of RNs that worked full-time in each group can be seen in Table 2. RNs’ median years since nursing certificate at baseline and post-intervention were 9.0 and 7.0 years for the intervention group and 7.0 and 6.0 years for the control group. The median years of experience for RNs at their current unit were 3.1 and 2.7 years for the intervention group and 3.7 and 2.0 years for the control group, at baseline and post-intervention, respectively. The percentage of RNs in the intervention group with an advanced educational level (master or specialist education) was 48.0 % at baseline and 46.0 % at post-intervention and 40.4 and 34.0 %, respectively, for the control group. RNs’ awareness of the CPGs was 68.5 % at baseline and 67.9 % post-intervention in the intervention group and 58.7 % at baseline and 77.5 % post-intervention for the control group (Table 2).

### PVC-related complications

There was no significant difference between the groups regarding the effect of the reminders on the proportion of reasons for removals due to PVC-related complications (*p* = 0.18, 95 % confidence interval (CI) −12.8 % to +2.3 %). The complication rate in the intervention group was 254 (40.6 %) at baseline and 259 (41.9 %) post-intervention and in the control group 292 (40.3 %) at baseline and 316 (46.9 %) post-intervention. The most frequent complication in the intervention group was infiltration followed by occlusion and signs and symptoms of thrombophlebitis, whereas occlusion followed by infiltration and thrombophlebitis was most frequent in the control group (Table 3).

**Table 3:** Reasons for removal due to PVC-related complications and elective reasons

	Intervention		Control	
	Baseline (*n* = 626)	Post-intervention (*n* = 618)	Baseline (*n* = 724)	Post-intervention (*n* = 674)
Reasons for removal of PVCs due to complications				
*n* (%)	254 (40.6 %)	259 (41.9 %)	292 (40.3 %)	316 (46.9 %)
95 % CI	36.7–44.5	38.0–45.8	36.8–44.0	43.1–50.7
Infiltration including extravasation, *n* (%)	105 (17.1)	108 (17.5)	73 (10.1)	103 (15.3)
Occlusion, *n* (%)	88 (14.1)	94 (15.2)	130 (18.0)	119 (17.7)
Sign and symptoms of thrombophlebitis, *n* (%)	37 (5.9)	34 (5.5)	63 (8.7)	67 (9.9)
PVC accidentally removed, *n* (%)	20 (3.2)	18 (2.9)	20 (2.8)	23 (3.4)
Suspicion of infection, *n* (%)	3 (0.5)	4 (0.6)	5 (0.7)	2 (0.3)
Wound (pressure wound), *n* (%)	1 (0.2)	1 (0.2)	1 (0.1)	2 (0.3)
Elective reasons for removal of PVC^a^				
*n* (%)	372 (59.4 %)	359 (58.1 %)	432 (59.7 %)	358 (53.1 %)
95 % CI	55.5–63.3	54.1–62.0	56.0–63.2	49.3–56.9

^a^Included completed treatment; the patient is bothered by the PVC, PVC re-sited in connection to blood sampling, or change to central venous access device and other reasons (e.g., location dependent, been inserted too long, and blood at insertion site)

### RNs’ adherence to the CPG for PVCs

There was no significant difference between the groups regarding RNs’ adherence to the CPG recommendations from baseline to post-intervention, as seen in Table 4: disinfection of hands OR = 2.05 (95 % CI 0.6–7.4), usage of disposable gloves OR = 0.96 (95 % CI 0.4–2.2), or daily inspection OR = 0.77 (95 % CI 0.4–1.7).

**Table 4:** RNs’ adherence to the CPG recommendations at baseline and post-intervention—logistic regression analysis

			Logistic regression	
CPG recommendations (total number)	Number of respondents	Adherence *n* (%)^a^	OR (95 % CI)^b^	*p* value
Disinfection of hands				
Intervention			2.05 (0.6–7.4)	0.270
Baseline (*n* = 108)	108	97 (89.8)		
Post-intervention (*n* = 106)	105	93 (88.6)		
Control				
Baseline (*n* = 104)	103	96 (93.2)		
Post-intervention (*n* = 102)	102	87 (85.3)		
Usage of disposable gloves				
Intervention			0.96 (0.4–2.2)	0.923
Baseline (*n* = 108)	108	80 (74.1)		
Post-intervention (*n* = 106)	105	76 (72.4)		
Control				
Baseline (*n* = 104)	103	71 (68.9)		
Post-intervention (*n* = 102)	102	70 (68.6)		
Daily inspection of PVC site				
Intervention			0.77 (0.4–1.7)	0.499
Baseline (*n* = 108)	108	58 (53.7)		
Post-intervention (*n* = 106)	103	58 (56.3)		
Control				
Baseline (*n* = 104)	102	47 (46.1)		
Post-intervention (*n* = 102)	102	55 (53.9)		

^a^Adherence = answered always

^b^OR for interaction effect of being in the intervention group post-intervention

### Context

Table 5 displays RNs’ mean scores for leadership, culture and evaluation, and the categorisation of these dimensions into high and low, as well as the allocation of RNs into four context groups. The categorisation of RNs’ mean scores into high or low was significantly different between the intervention and control units for culture (*p* = 0.037), with RNs in the intervention group scoring higher. The distribution of RNs, at all units into high or low, was equal for leadership, while the majority were high for culture and low for evaluation. There was a significant difference (*p* = 0.013) in the distribution over the four context groups between the intervention and control units. An almost equal number of RNs at all units were distributed into either high or low context, while a majority of the RNs at the intervention units were distributed into moderately high context followed by moderately low context, and the pattern for the control units was reversed.

**Table 5:** Context scorings in intervention and control units at baseline

	Intervention units	Control units	Fisher’s exact test
	RNs, *n* (%)	RNs, *n* (%)	*p* value
Context dimensions	RNs = 108	RNs = 104	0.890
Leadership	104 (96.3)	102 (98.1)	
Mean (SD)	3.4 (0.8)	3.6 (0.8)	
High^a^	52 (50.0)	50 (49.0)	
Low^b^	52 (50.0)	52 (51.0)	
Culture	104 (96.3)	103 (99.0)	0.037
Mean (SD)	4.0 (0.5)	3.8 (0.7)	
High^a^	85 (81.7)	71 (68.9)	
Low^b^	19 (18.3)	32 (31.1)	
Evaluation	105 (96.3)	100 (96.2)	0.759
Mean (SD)	3.1 (0.7)	3.0 (0.9)	
High^a^	29 (27.6)	30 (30.0)	
Low^b^	76 (72.4)	70 (70.0)	
Context groups^c^	RNs = 101	RNs = 97	
High context^d^	14 (13.9)	22 (22.7)	
Moderately high context^e^	44 (43.6)	22 (22.7)	
Moderately low context^f^	31 (30.7)	34 (35.1)	
Low context^g^	12 (11.9)	19 (19.6)	

^a^High = individual mean score >3.5

^b^Low = individual mean score ≤3.5

^c^Significant difference (*p* = 0.013) in the distribution of the different context groups between intervention and control units

^d^High scores on all context dimensions

^e^High on two dimension and low on one

^f^High on one dimension and low on two

^g^Low scores on all dimensions

## Discussion

The results showed no significant effects of implementing the CPG recommendations in the format of reminders in the EPR on PVC-related complications or on RNs’ adherence to the CPG. Also, there was no considerable difference in the RNs’ scoring of the context between the groups. The context in both groups was predominantly perceived as moderately low to moderately high, indicating that the conditions might not have been optimal for a successful implementation.

The complication rate at baseline and post-intervention in both groups can be considered as high, as around 40 % of the recorded PVCs were removed due to signs and symptoms of complications. The number of PVCs that were removed due to suspicion of infection was low at baseline and post-intervention in both groups, which might be related to the rather high adherence to disinfection of hands. The adherence to the CPG recommendations was quite stable in both groups throughout the study period, but the adherence for disposable gloves and daily inspection showed a greater improvement potential compared to disinfection of hands. As daily inspection of the PVC site is an essential procedure in order to detect early signs or symptoms of PVC-related complications, the low adherence to this recommendation is of concern.

There are few studies on the effects of computerised reminders in nursing, and to our knowledge, there are no other studies on the effect of computer reminders on RNs adherence to a CPG for PVCs and on patient outcomes related to PVCs. Therefore, we will discuss our findings in relation to research on computerised support interventions in general that is of relevance for our findings. A systematic review concludes that CDSSs that advise health care professionals automatically in the workflow at the point of care were not significantly associated with success [36]. However, systems that provided advice to both patients and health care professionals were more likely to succeed as well as CDSS that required the user to provide reason for deviating from recommendations. The authors of the review conclude that interactive CDSS seems to be more successful than CDSS that simply provide advice to health care professionals. The intervention used in this study involved reminders that integrated recommendations from the CPG into clinical practice by a relatively simple instrumental innovation. A recent overview of systematic reviews found no compelling evidence that multifaceted implementation interventions would be more effective in changing health care professionals’ behaviours compared to single component interventions [37]. But the authors argue that it might be appropriate to design single or less complex multifaceted implementation interventions that are more tailored to overcome contextual barriers and improve the targeted behaviour. Studies that have showed some positive outcomes concerning the use of CDSS in nursing practice in recent years have, in addition to CDSS, also included educational sessions [18, 38, 39] and/or feedback [40]. The outcome of the present study might have benefitted from using facilitators to overcome the barriers of rigorous PVC documentation and regular feedback on items such as PVC-related complications, for example.

The development and integration of computer reminders are dependent on the existing EPR systems’ usability and flexibility, which might hinder an optimal integration. RNs at the intervention units were only exposed to the reminders when recording in the template designated for PVCs, which meant that RNs who did not use the PVC template in the EPR were not exposed to the reminders. While we could not control where RNs recorded PVCs, we chose to only retrieve data from the PVC template to make sure that the included PVCs were documented by RNs in the intervention group that had been exposed to the reminders. Another consideration is that the fields in the template for recording PVC insertion (date, reason, attempts, side, site, and size) were mandatory; in other words, they had to be completed to save and close the template. Due to the EPR design restrictions, the fields relating to PVC removal (date, cause, and daily inspections) could not be made mandatory. With the range of 40.3–49.1 % missing data regarding reasons for removal, we can assume that RNs were more exposed to the reminders that were activated when recording PVC insertion but less likely to see the activated reminders regarding removal and inspection.RNs perception and experiences of using the computer reminders at the interventions units are described in a separate article [41]. The CPG and the reminder concerning daily inspection of the PVC site seemed to have little effect on RNs’ behaviours, implying that early sign and symptoms of complications may have gone unnoticed.

In a study on an implementation support intervention like this, an analysis of the context at participating units was considered to be of importance. As suggested in the PARIHS framework, the more favourable the context, the better the conditions for successful implementation [6], meaning that high ratings of context would be favourable for the implementation of the CPG recommendations. There was a significant difference in scorings between intervention and control in one of three context dimensions. However, as most RNs scored their context as moderately low or moderately high, it indicates that the conditions for a successful implementation to occur were less optimal. A majority of the RNs in both groups scored the culture at their units as high, while the mean scores for leadership were almost equally distributed between high and low for both groups. Evaluation was scored low by a majority of the RNs in both groups. A pilot study with a leadership intervention designed to influence RNs’ use of guideline recommendations concerning diabetic foot ulcers suggests that the unit manager’s involvement played a role in initiating and enabling a change towards evidence-based practice [42]. The authors argue that further understanding of how context interacts with leaders’ ability to influence guideline adherence is needed, as well as the knowledge of what kind of organisational structures and processes are required to support nurse managers and clinical leaders in conducting relevant evaluations, such as with auditing and feedback. A previous study by our group [33] showed a significant relationship between RNs’ scores of the evaluation dimension and adherence to the recommendation on daily inspection of PVC sites. We were, however, not able to investigate the direction of the relationship; in other words, if RNs that received regular feedback concerning nursing procedures performed daily inspection of PVC sites more often or if RNs that regularly carried out daily inspections of PVC sites received more feedback.

The PARIHS framework suggests that successful implementation is more likely to occur when evidence and context are considered high. Evidence should then have a sound research base and coincide with clinical and patient experiences [6]. The research base for the CPG recommendations could most likely have been more robust. Out of the 11 references that were included in the CPG, five were studies conducted in paediatric settings and none of these were referred to when recommending the choice of PVC site, size, or signs and symptoms of PVC-related complications. Professional experience was referred to when recommending PVCs sites that should be avoided. One can only speculate if the impact would have been different if the CPG could have been based on more rigorous evidence. Unfortunately, we do not have any data on how RNs perceived the CPG for PVCs, but it is a concern that approximately one third of the RNs (varying from 22.5 to 41.3  % between groups and baseline to post-intervention) stated that they did not know about the CPGs for venous catheters (Table 2).

### Methodological considerations

The primary outcome, PVC-related complications, was based on individual RNs’ observations and recordings, and we cannot verify if the recorded reason for removal was the accurate reason for removal or the exact number of real PVC-related complications. Consideration must also be given to the fact that the documentation might have improved over time, which may have resulted in an increase in recorded PVC-related complications. These two considerations, however, should not have affected the outcome as the shortcoming of the PVC recording and related complications should have been equally distributed in the intervention and control group due to the randomisation. It should be mentioned that we do not report the possible patient-related factors (i.e., the child’s diagnosis/condition and type of pharmacological treatment administered via the PVC) that may have influenced the complication rate. The signs and symptoms of PVC-related complications in children are often nonspecific and sometimes interrelated. The signs and symptoms of infiltration including extravasation and occlusion can be similar, and we cannot guarantee that the documented complications were accurate.

It should be mentioned that an analysis of patients’ ages between the two groups based on the division of neonatal patients was performed as these patients are more likely to have PVC-related complications compared to patients at the paediatric units [43]. PVCs recorded at neonatal units represented 58.7 % (*n* = 149) of the complications in the intervention group at baseline and 53.7 % (*n* = 139) post-intervention. Ten percent (*n* = 30) of the PVC-related complications in the control group related to neonatal units at baseline and 5.4 % (*n* = 17) at post-intervention. Age, based on division of neonatal patients, was not seen as a bias as the intervention group had more PVC-related complications among neonatal patients, compared to the control group, but the overall complication rate was below the occurrence found in the control group.

The second outcome, RNs’ adherence to the CPG was based on self-reported data, which imply a risk for social desirability leading to over-reporting of preferred procedures for the management of PVCs [44]. However, it does not seem that social desirability has affected the outcome to a high extent, as ratings of adherence to daily inspection of the PVC site were low. The computer reminders appeared when RNs were recording, and thus, they were exposed to the reminder after the targeted procedure. However, as the insertion, management, assessment, and documentation of PVCs are common daily procedures among Swedish paediatric RNs, we assumed that the reminders likely would impact RNs behaviour in forthcoming PVC management.

The PARIHS framework was used to structure this study. In hindsight, it would have been an advantage to map the context of the setting before designing the intervention. By using a theoretical framework emphasising the importance of evidence and context, the opportunities to reflect on why the intervention had no impact increased, as well as better insights and future intervention ideas could be derived.

## Conclusions

The present cluster randomised controlled study implemented recommendations from a CPG in the format of reminders integrated into a PVC template in the EPR system. The results showed no significant effects on PVC-related complications or on RNs’ adherence to the CPG recommendations. The context in both groups was assessed to primarily vary from moderately low to moderately high, indicating that a successful implementation of the CPG recommendations in the format of reminders was probably less likely to occur. The intervention might have benefitted from a more tailored intervention that targeted specific barriers at the inpatient units, for example, the low frequency of recorded reasons for removal and daily inspection of PVC site, as well as the lack of regular feedback to RNs.
